# Transcriptome analysis of a newly established mouse model of *Toxoplasma gondii* pneumonia

**DOI:** 10.1186/s13071-022-05639-3

**Published:** 2023-02-08

**Authors:** Long Cheng, Sajid Ur Rahman, Hai-Yan Gong, Rong-Sheng Mi, Yan Huang, Yan Zhang, Ju-Liang Qin, Cheng-Cong Yin, Min Qian, Zhao-Guo Chen

**Affiliations:** 1 Shanghai Key Laboratory of Regulatory Biology, Institute of Biomedical Sciences and School of Life Sciences, East China Normal University, Shanghai, China; 2 Key Laboratory of Animal Parasitology of Ministry of Agriculture and Rural Affairs, Laboratory of Quality and Safety Risk Assessment for Animal Products on Biohazards (Shanghai) of Ministry of Agriculture and Rural Affairs, Shanghai Veterinary Research Institute, Chinese Academy of Agricultural Sciences, Shanghai, 200241 China; 3 Department of Food Science and Engineering, School of Agriculture and Biology, Shanghai Jiao Tong University, Shanghai, 200240 China

## Abstract

**Background:**

Toxoplasmosis is a zoonotic parasitic disease caused by *Toxoplasma gondii*. *Toxoplasma gondii* infection of the lungs can lead to severe pneumonia. However, few studies have reported *Toxoplasma* pneumonia. Most reports were clinical cases due to the lack of a good disease model. Therefore, the molecular mechanisms, development, and pathological damage of *Toxoplasma* pneumonia remain unclear.

**Methods:**

A mouse model of *Toxoplasma* pneumonia was established by nasal infection with *T. gondii*. The model was evaluated using survival statistics, lung morphological observation, and lung pathology examination by hematoxylin and eosin (H&E) and Evans blue staining at 5 days post-infection (dpi). Total RNA was extracted from the lung tissues of C57BL/6 mice infected with *T. gondii* RH and TGME49 strains at 5 dpi. Total RNA was subjected to transcriptome analysis by RNA sequencing (RNA-seq) followed by quantitative real-time polymerase chain reaction (qRT–PCR) validation. Transcript enrichment analysis was performed using the Gene Ontology (GO) and Kyoto Encyclopedia of Genes and Genomes (KEGG) databases to assess the biological relevance of differentially expressed transcripts (DETs).

**Results:**

C57BL/6 mice infected with *T. gondii* via nasal delivery exhibited weight loss, ruffled fur, and respiratory crackles at 5 dpi. The clinical manifestations and lethality of RH strains were more evident than those of TGME49. H&E staining of lung tissue sections from mice infected with *T. gondii* at 5 dpi showed severe lymphocytic infiltration, pulmonary edema, and typical symptoms of pneumonia. We identified 3167 DETs and 1880 DETs in mice infected with the *T. gondii* RH and TGME49 strains, respectively, compared with the phosphate-buffered saline (PBS) control group at 5 dpi. GO and KEGG enrichment analyses of DETs showed that they were associated with the immune system and microbial infections. The innate immune, inflammatory signaling, cytokine-mediated signaling, and chemokine signaling pathways displayed high gene enrichment.

**Conclusion:**

In this study, we developed a new mouse model for *Toxoplasma* pneumonia. Transcriptome analysis helped to better understand the molecular mechanisms of the disease. These results provided DETs during acute *T. gondii* lung infection, which expanded our knowledge of host immune defenses and the pathogenesis of *Toxoplasma* pneumonia.

**Graphical Abstract:**

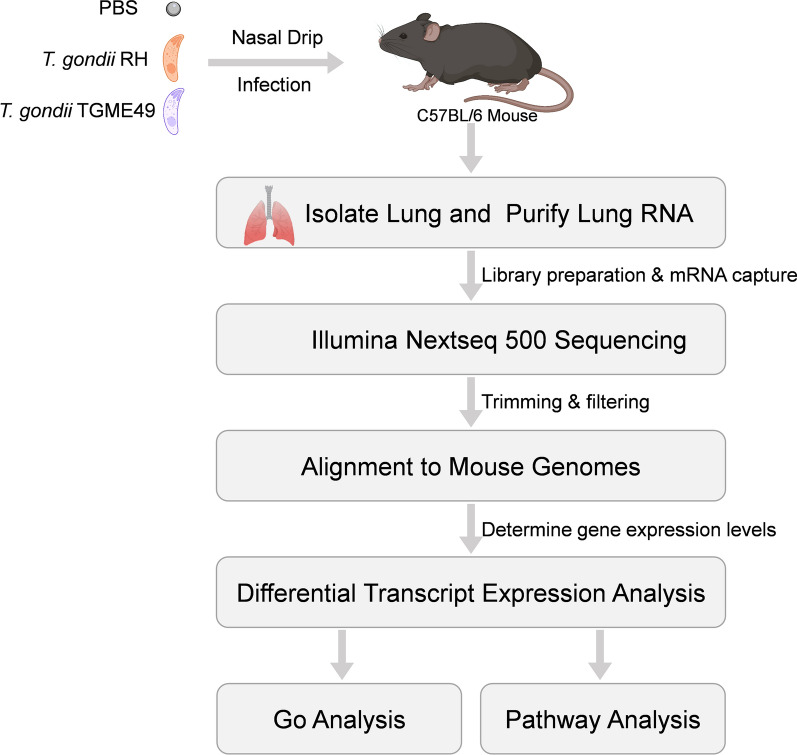

**Supplementary Information:**

The online version contains supplementary material available at 10.1186/s13071-022-05639-3.

## Background

Approximately 25–30% of the world's population is believed to be infected with *Toxoplasma gondii* [1]. *Toxoplasma gondii* can be divided into three classical clonal lineages (types I, II, and III) according to its virulence in mice [2]. Infection with *T. gondii* can cause fever and flu-like symptoms in individuals with normal immune functions [3]. *Toxoplasma gondii* infects patients with immunodeficiency, such as those with acquired immunodeficiency syndrome (AIDS) and organ transplant patients, leading to consequences that can be fatal in severe cases. Infection with *T. gondii* can also cause miscarriage in pregnant women [4] and infect many organs in the human body, such as the brain, lungs, and heart [5].

The host immune system defense against *T. gondii* infection is primarily based on the immune response mediated by Th1 cells, which produce high levels of interleukin-12 (IL-12) and interferon-gamma (IFN-γ) [6]. After infection with *T. gondii*, innate immune cells (including dendritic cells, macrophages, and neutrophils) migrate to the sites of infection and recognize *T. gondii* through toll-like receptors (TLRs) and secrete IL-12. IL-12 stimulates CD^4+^ and CD^8+^T cells to produce IFN-γ [7]. IFN-γ limits parasite proliferation and infection progression through various intracellular mechanisms, such as stimulation of guanylate-binding proteins (GBPs) and immunity-related GTPases (IRGs), to degrade parasitophorous vacuoles (PV) [8]. IL-12 and IFN-γ are key cytokines involved in tachyzoite clearance during acute infections. The anti-inflammatory cytokines IL-4, IL-10, IL-13, IL-27, and transforming growth factor beta (TGF-β) are responsible for minimizing the damage caused by excessive inflammatory responses in the immune system [9]. The long evolution between *T. gondii* and its mammalian host has led to three clear pathways that enable cells to recognize and destroy *T. gondii*: the TLR signaling pathway, inflammasome, and IFN-induced GTPases [10].

In recent years, *Toxoplasma* pneumonia has become an important opportunistic infection in patients with AIDS [11], accounting for an estimated 4% of the total *T. gondii* infection. The lung is the second or third most important site of *T. gondii* infection after the central nervous system (CNS) [5]. The diagnosis of *Toxoplasma* pneumonia is challenging because the clinical symptoms of this disease are not specific and are similar to those of other types of pneumonia. *Toxoplasma gondii* infection of the lungs can lead to severe pneumonia. However, there are very few reports on *Toxoplasma* pneumonia; most reports are related to clinical cases [12, 13]. This is due to a lack of a good disease model for *Toxoplasma* pneumonia. As a result, the molecular mechanisms underlying the occurrence, development, and pathological damage of *Toxoplasma* pneumonia remain unclear.

In this study, a mouse model of *Toxoplasma* pneumonia was successfully developed via nasal droplet infection with *T. gondii*. Transcriptome analysis was also performed to better understand the immune system response and defense response of mice to the protozoan during *Toxoplasma* pneumonia.

## Methods

### Cell and *Toxoplasma gondii* culture

Vero cells and *T. gondii* were preserved in the laboratory. Vero cells were cultured in high-glucose Dulbecco’s modified Eagle’s medium (Sigma-Aldrich, St. Louis, MO, USA) containing 100 U/ml penicillin, 100 U/ml streptomycin, and 2% (v/v) fetal bovine serum (Gibco, New York, NY, USA). The cells were incubated at 37 °C in a humidified atmosphere containing 5% CO_2_. *Toxoplasma gondii* RH and TGME49 strains were obtained by passaging into Vero cells. When 90% of the Vero cells were lysed, tachyzoites were collected and purified using Percoll (GE Healthcare, Boston, MA, USA) density gradient centrifugation.

### Lung infection of mice with *Toxoplasma gondii*

Six-week-old female C57BL/6 mice weighing 21 g were used in this study. The mice were anesthetized using intraperitoneal injection of 0.7 ml 10% chloral hydrate. After anesthetization, mice were infected with nasal drops/1000 *T. gondii* tachyzoites in phosphate-buffered saline (PBS). *Toxoplasma gondii* diluted in PBS had a total volume of less than 35 μl per mouse; the same volume of PBS was used as a control.

### Paraffin sections and hematoxylin and eosin staining

After mice were infected with *T. gondii*, at 5 days post-infection (dpi), they were killed by cervical dislocation. The chests were opened using scissors and the lung tissues were separated. The dissected mouse lung tissues were placed in 4% paraformaldehyde and fixed overnight at 25 °C. After gradient dehydration in ethanol, transparency in xylene, and overnight immersion in paraffin, tissues were cut into 5-μm-thick slices. Before hematoxylin and eosin (H&E) staining, the samples were deparaffinized and placed in xylene I, xylene II, 50% (v/v) xylene/ethanol, and 100, 90, 75, and 50% ethanol for 3 min. Finally, the samples were stored in pure water until subsequent staining. H&E staining was performed using an H&E kit (Servicebio, Wuhan, China) according to the manufacturer's instructions.

### Detection of *Toxoplasma gondii* in lung tissues

Approximately 25 mg of lung tissue was transferred to a 1.5 ml sterile centrifuge tube. Total DNA was extracted using a TIANamp genomic DNA kit (Tiangen, Beijing, China) according to the manufacturer's instructions. A 389-base-pair (bp) fragment of the *T. gondii* internal transcribed spacer (*ITS*) gene was amplified using polymerase chain reaction (PCR) with the primers ITS-F (5′-GAT TTG CAT TCA AGA AGC TGA TAG TAT-3′) and ITS-R (5′-AGT TAG GAA GCA ATC TGA AAG CAC ATC-3′). Positive and negative controls were included for each PCR. A was used for PCR amplification. PCR reactions contained 12.5 μl 2× Taq PCR MasterMix II (Tiangen, Beijing, China), including 1 μM of each primer and 1 μl of each individual DNA sample in a total reaction mixture of 25 μl. A total of 30 cycles were carried out, each one consisting of 94 °C for 30 s, 55 °C for 30 s, and 72 °C for 30 s, with an initial cycle starting at 94 °C for 2 min and a final extension at 72 °C for 5 min. The PCR products were examined using gel electrophoresis on a 1% agarose gel containing 4S Green Plus Nucleic Acid Stain (Sangon Biotech, Shanghai, China).

### Lung Evans blue staining

At 5 dpi, mice were injected with 1% Evans blue dye in PBS solution (100 μl) via the tail vein. After 3 h, mice were euthanized by cervical dislocation and immediately subjected to cardiac perfusion. The thoracic cavities were opened, the left atrial appendages were transected, and the lavage pump needle was inserted into the left ventricles and flushed with PBS until the effluent was colorless. The whole lung was weighed. The right lung was excised and transferred to an 1.5 ml sterile centrifuge tube containing 500 μl of formamide and placed in a 50 °C water bath for 24 h. The lung samples were then removed, and the sterile centrifuge tubes were centrifuged at 2000 rpm for 5 min. The supernatants were collected to measure the absorbance at 620 nm. A standard curve was prepared by plotting the average of each Evans blue standard versus its concentration in ng/ml. The standard curve was used to determine the Evans blue concentration in each unknown sample.

### Total RNA extraction and sequencing

Total RNA was extracted from the lungs using TRIzol reagent (Invitrogen, Carlsbad, CA, USA) according to the manufacturer's instructions. The quantity and quality of the RNA were determined using an Agilent 2100 Bioanalyzer according to the manufacturer’s instructions. Total RNA was isolated using poly-T oligo-conjugated magnetic beads. Mitochondrial RNA (mRNA) was transcribed into complementary DNA (cDNA) using the PrimeScript™ RT reagent kit with gDNA Eraser (TaKaRa, Dalian, China) according to the manufacturer's instructions. Mouse lung transcriptomic library construction and RNA sequencing (RNA-Seq) were performed by the NovelBio Corporation (Shanghai, China).

### Sequencing, quality, and mapping of reads

The reads were trimmed to remove adaptor primers, low-quality reads, and very short (< 50 nucleotides [nt]) reads. The quality of RNA-seq was checked using the quality scores Q20 and Q30. The clean reads were mapped against the mouse reference genome using HISAT2 software. The reads per kilobase per million mapped reads (RPKM) method was used to calculate the relative gene expression.

### Bioinformatics analysis of the differentially expressed genes (DEGs)

DESeq2 software was used to determine gene expression and identify differentially expressed transcripts (DETs) between the *T. gondii* RH-infected and PBS control, *T. gondii* TGME49-infected and control, and *T. gondii* RH-infected and *T. gondii* TGME49-infected groups. The false discovery rate (FDR) was used to correct multiple hypothesis testing *P*-values. Genes with FDR-adjusted *P*-values of Fisher’s exact test ≤ 0.05 and |log2(fold change)|≥ 0.585 were deemed as DETs. Functional annotation and pathways involving the DETs were analyzed using Gene Ontology (GO) and Kyoto Encyclopedia of Genes and Genomes (KEGG) pathway enrichment analyses.

### Quantitative real-time polymerase chain reaction

Reliability testing of RNA-seq data was performed using quantitative real-time polymerase chain reaction (qRT–PCR). Eleven DETs were selected: *Tlr7*, *TLR9*, nucleotide-binding oligomerization domain-containing protein 2 (*Nod2*), interferon gamma (*Ifng*), interferon regulatory factor 5 (*Irf5*), C–C motif chemokine ligand 2 (*Ccl2*), MHC class II transactivator (*Ciita*), nucleotide-binding domain-like receptor protein 1 (*Nlrp1*), *Cd36*, *Il11*, and *Ccl24*. Glyceraldehyde-3-phosphate dehydrogenase (*Gapdh*) served as the reference gene. Total RNA was extracted from mouse lungs using TRIzol and reverse-transcribed into cDNA using a reverse transcription kit following the manufacturer’s instructions (Promega, Madison, WI, USA). cDNA was stored at −20 °C until use. Detailed information on all primers is shown in Additional file 1: Table S1. The qRT–PCR conditions were as follows: amplification at 98 °C for 15 s, followed by 40 cycles of denaturation at 98 °C for 10 s, and annealing at 60 °C for 15 s. Melting curve analysis ranged from 72 °C to 95 °C to ensure that specific products were amplified in each qRT–PCR reaction.

## Results

### Establishment of mouse *Toxoplasma* pneumonia model

**Fig. 1:**
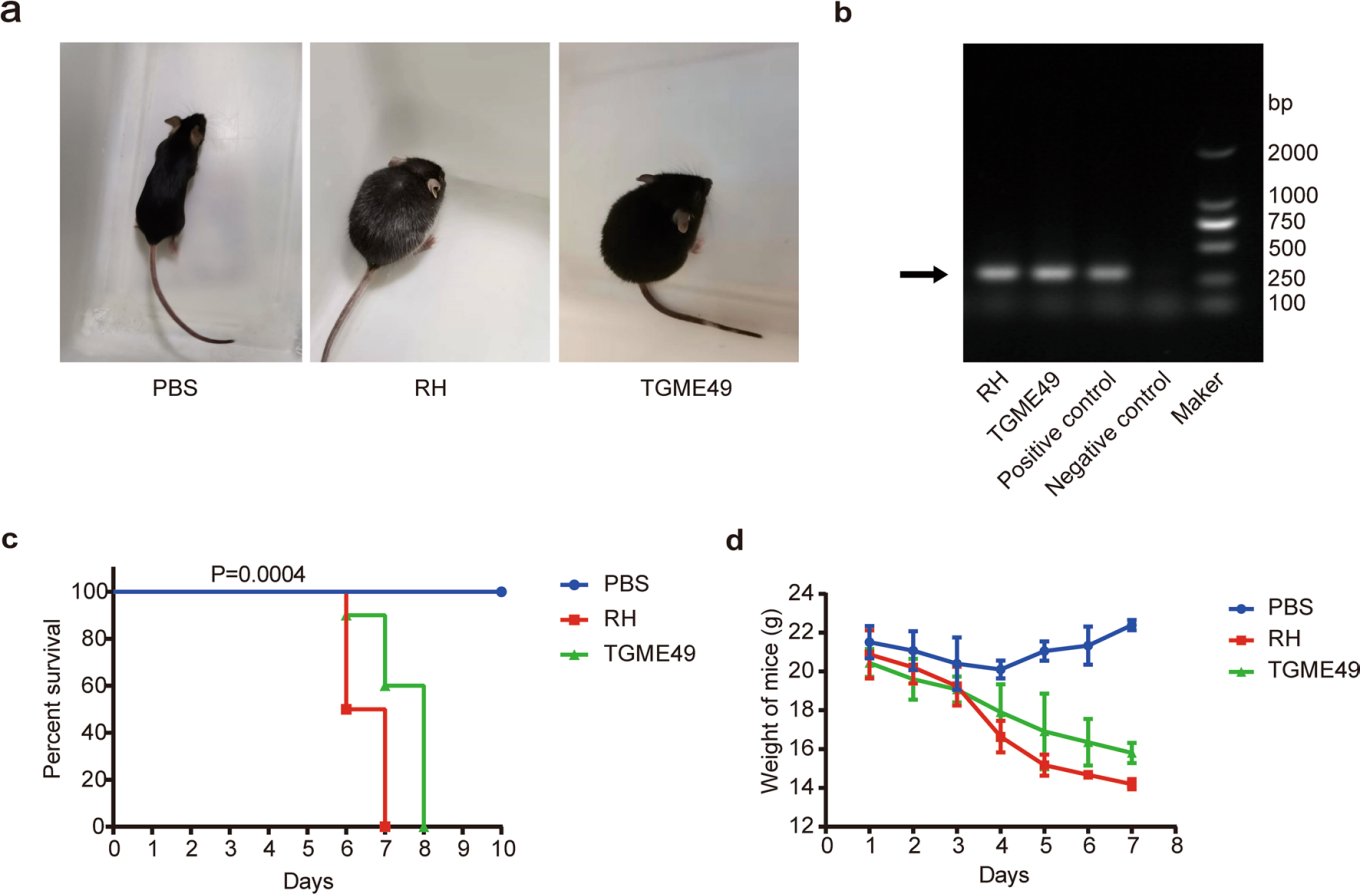
*Toxoplasma gondii* lung infection reduced survival and weight loss in mice. **a** Clinical symptoms of infected mice. **b** Survival rate of mice after *T. gondii* infection. **c** Weight of mice after *T. gondii* infection

A mouse *Toxoplasma* pneumonia model was successfully constructed by nasal infection with *T. gondii* tachyzoites. The data in Fig. 1a show that the mice were susceptible to the *T. gondii* RH and the *T. gondii* TGME49 strains, and manifested similar diseases with often fatal outcomes, such as body weight loss, ruffled fur, and respiratory failure. *Toxoplasma gondii* in the lung tissue was detected using PCR. The results showed that the *T. gondii* RH and TGME49 strains successfully infected the lungs of the mice (Fig. 1b). Survival analysis revealed that all the mice in the PBS control group survived. However, *T. gondii* lung infection resulted in the death of mice; the mortality rate was 100%. The group infected with the *T. gondii* RH strain started dying at 6 dpi, and all the mice had died at 7 dpi. The group infected with *T. gondii* TGME49 started dying at 7 dpi, and all the animals had died at 8 dpi (Fig. 1c). The average weight of mice in the group infected with the *T. gondii* RH strain decreased at 4 dpi and was more significant than that in the TGME49 infection group. In contrast, the average weight of the mice was marginally increased in the PBS control group compared with before the test (Fig. 1d).

### Pulmonary vascular permeability assessment and lung wet weight measure

**Fig. 2:**
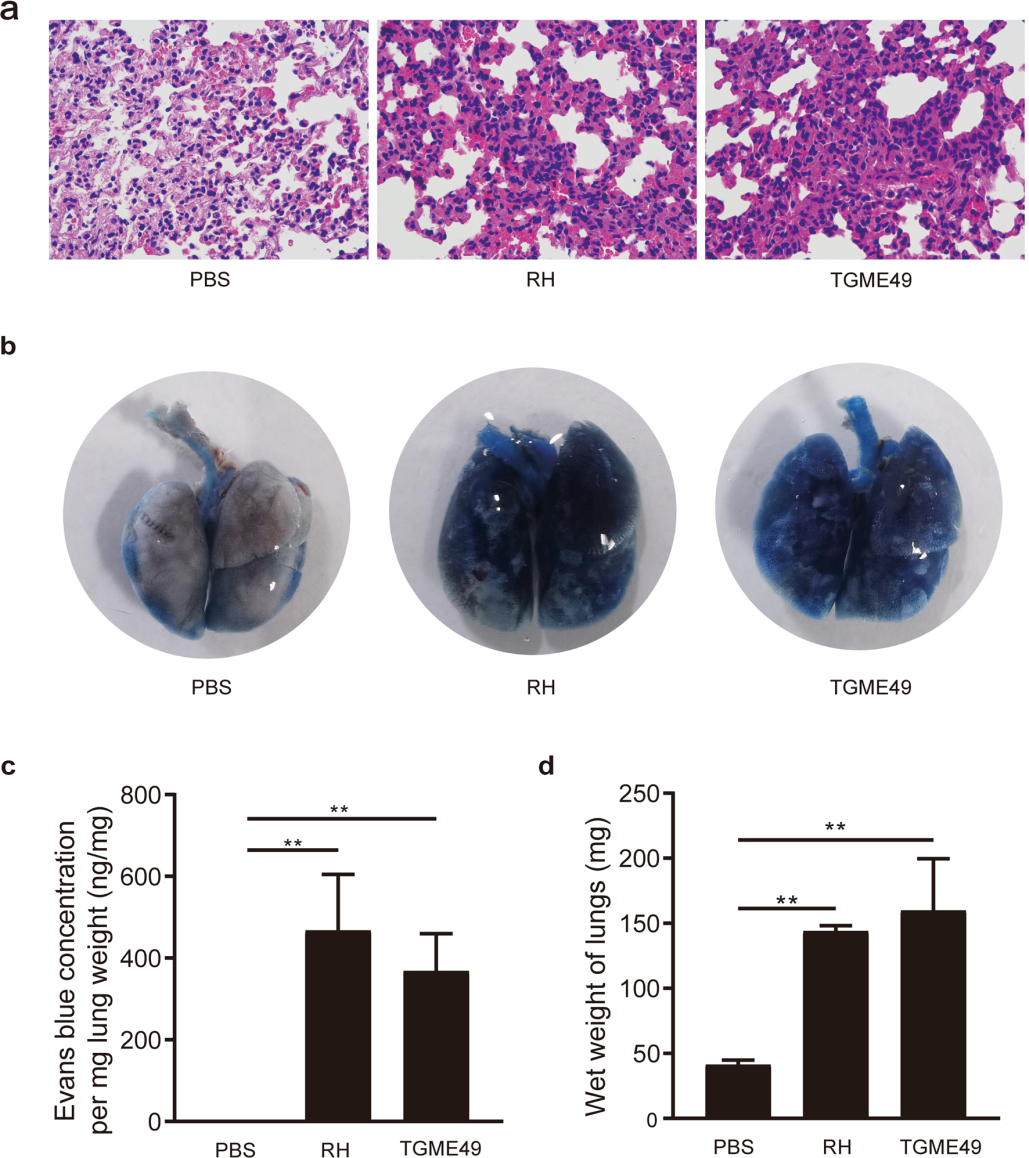
Structural changes in the lung infected with *T. gondii* lead to severe disruption of the air–blood barrier and severe pulmonary edema in mice. **a** H&E staining of lung paraffin section was observed 5 days post-*T. gondii* infection. **b** Lung Evans blue staining was observed 5 days post-*T. gondii* infection. **c** Lung Evans blue OD620 absorbance. **d** Lung wet weight at 5 days post-*T. gondii* infection. *N* = 4. One-way analysis of variance (ANOVA) was used for statistical analysis of data, where ** represents *P* < 0.01

Evans blue dye was injected into the tail veins of mice to determine the permeability of the blood-air barrier of the lungs at 5 dpi. Compared with the control group, mice in the *T. gondii* infection groups showed homogeneous light pink staining in the alveolar cavities, thickening of the alveolar walls, desquamation of the lung epithelium, and severe lymphocytic infiltration (Fig. 2a). The lung tissues of the PBS group did not become significantly blue. The Evans blue concentration per mg lung weight was ~ 0.00 ng/mg. The lungs of the mice in the *T. gondii* RH and TGME49 infection groups were distinctly blue in color. Per mg lung weight Evans blue concentrations were 467.97 ng/mg in the *T. gondii* RH infection group and 368.13 ng/mg in the *T. gondii* TGME49 infection group, which were significantly different from that of the control group (*P* < 0.01) (Fig. 2b, c). The average lung weight in the PBS control group was 41.38 mg. However, the average lung weight of the *T. gondii* RH and TGME49 infection groups were 144.17 and 159.85 mg, respectively, which were significantly higher than that of the PBS group (*p* < 0.01) (Fig. 2d).

### Quality of transcriptome analysis

Transcriptome analysis of the mouse lungs revealed that 97% of reads had good-quality scores > Q20, 93% had good-quality scores > Q30, and 60 million clean reads were generated in each sample (Table 1).

**Table 1:** Characteristics of the RNA-seq results obtained in the present study

Groups	Sample	All reads	Cleans reads	Clean bases	Mapped rate (%)	Q20 rate (%)	Q30 rate (%)	GC content (%)
Control	PBS 1	53,645,848	53,100,000	7,756,700,000	96.50	97.60	93.21	49.66
	PBS 2	40,765,488	40,362,000	5,924,500,000	96.50	97.61	93.22	49.63
	PBS 3	58,661,660	58,067,000	8,522,200,000	96.60	97.72	93.48	50.08
RH	RH 1	51,686,308	51,297,794	7,576,799,674	96.00	97.76	93.54	48.50
	RH 2	59,174,998	58,635,374	8,583,541,805	95.60	97.74	93.49	49.58
	RH 3	58,817,144	58,236,720	8,521,935,992	95.60	97.64	93.32	49.05
TGME49	TMGE49 1	58,164,082	57,647,000	8,451,100,000	95.70	97.74	93.49	49.29
	TGME49 2	52,661,872	52,197,000	7,637,200,000	95.90	97.64	93.32	48.43
	TGME49 3	36,605,664	36,289,000	5,354,300,000	96.00	97.55	93.09	49.39

### Differentially expressed transcripts

**Fig. 3:**
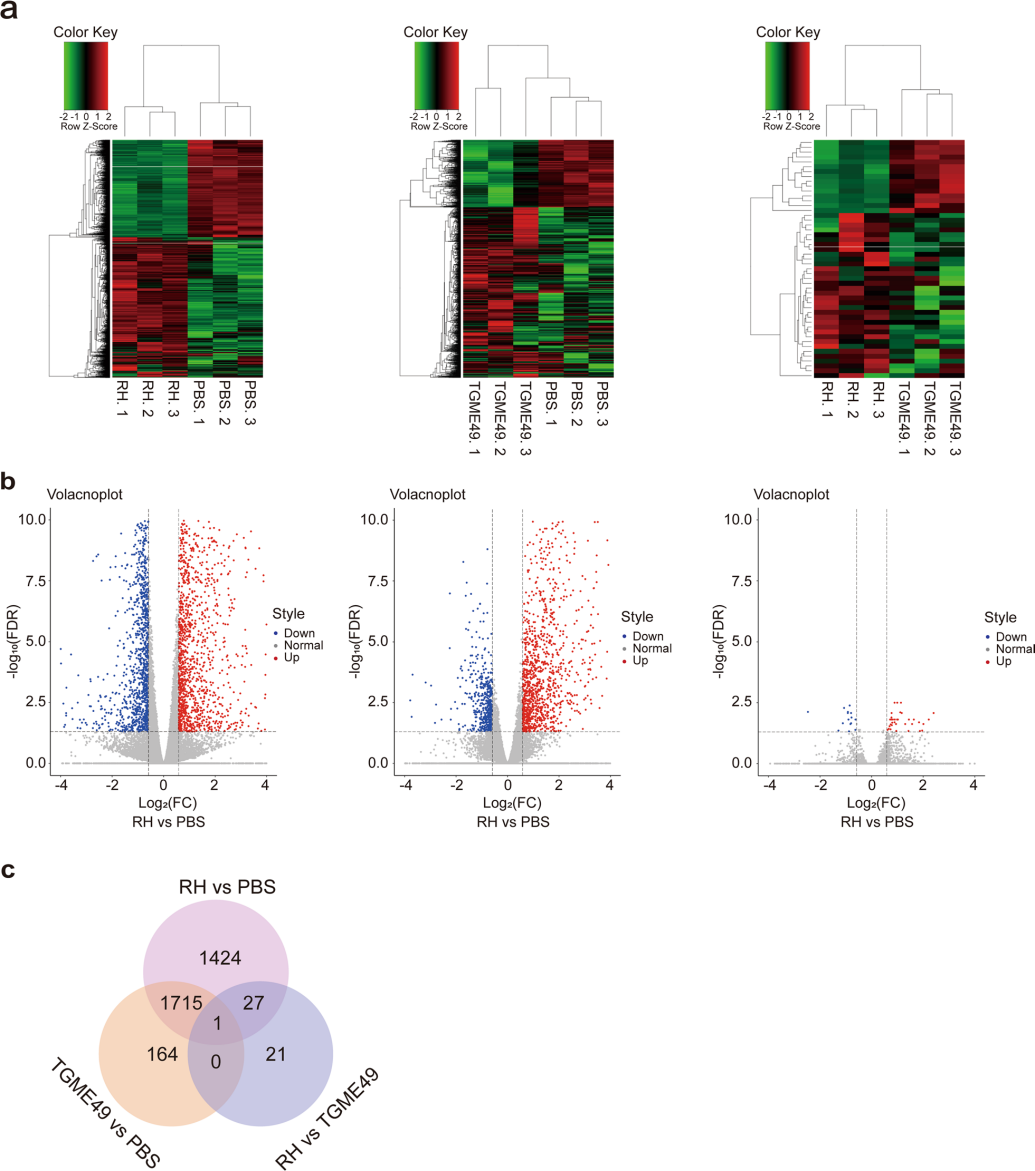
RNA sequencing and differential expression analysis. **a** Heat maps showed the hierarchical clustering of differentially expressed transcripts in *T. gondii* RH and TGME49 infected mice lung. Each mouse group included three biological replicates. **b** Volcano plot showed the differentially expressed transcripts, including upregulated and downregulated transcripts in *T. gondii* RH and TGME49 infected mice lungs. **c** Venn diagram showing shared and unique expression patterns between infection of *T. gondii* strains

The RNA-seq results showed a clear separation between *T. gondii* infection groups and the PBS control group. All DETs (adjusted *P*-value < 0.05; |log2FC|> 0.585) between biological replicates in the *T. gondii* infection groups or PBS control group presented low variation. A total of 3167 DETs (1871 upregulated and 1296 downregulated) were detected in the lungs of mice infected with *T. gondii* RH strain compared with the PBS control (Fig. 3a, b and Additional file 2: Table S2). In contrast, 1880 DETs (1346 upregulated and 534 downregulated) were detected in the lungs of mice infected with *T. gondii* TGME49 strain compared with the PBS control (Fig. 3a, b and Additional file 3: Table S3). As shown in the Venn diagram, two pairwise comparisons of *T. gondii* RH and TGME49 DETs, where the DETs were present during *T. gondii* RH and TGME49 infections in lung tissues, indicated that 1715 DETs (1284 upregulated and 431 downregulated) were common between the two strains. In addition, 1424 DETs (573 upregulated and 851 downregulated) were unique to the RH infection group, and 164 DETs (62 upregulated and 102 downregulated) were unique to the TGME49 infection group (Fig. 3c). The qRT-PCR results of eleven DETs that were randomly selected showed simliar tendency compare with RNA-seq data (Additional files 4: Figure S1).

### Disease pathway enrichment analysis of DETs

**Fig. 4:**
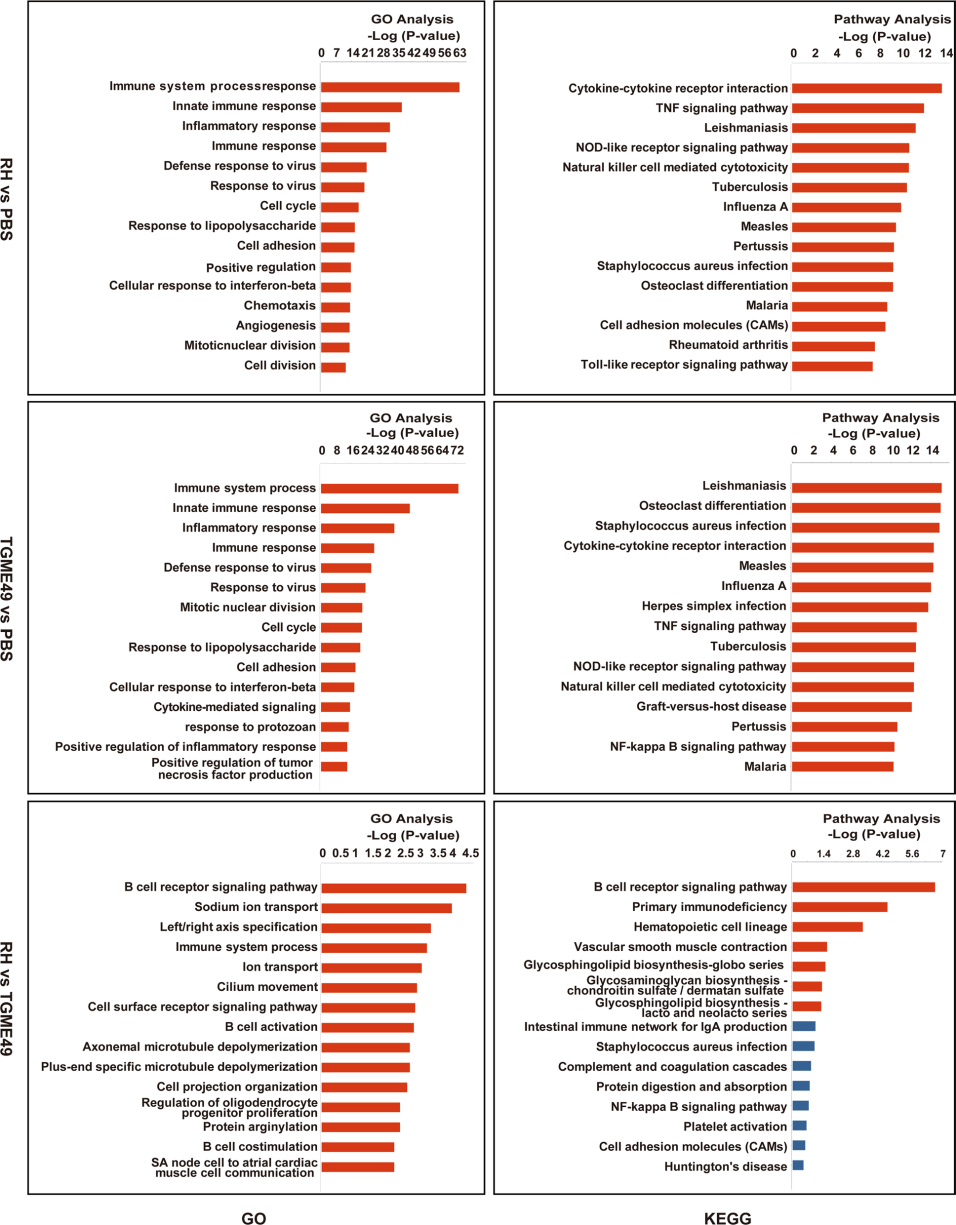
The top 15 biological process GO and KEGG pathways of the DETs detected in the mice lungs infected with *T. gondii* RH and TGME49 strains

To identify functional genes potentially associated with *Toxoplasma* pneumonia, GO and KEGG analyses were performed, as shown in Fig. 4 and Additional file 5: Table S4, Additional file 6: Table S5, Additional file 7: Table S6, Additional file 8: Table S7. GO terms were divided into three categories: biological process (BP), cellular component (CC), and molecular function (MF). GO analysis of the BP revealed that DETs were significantly enriched in the *T. gondii*-infected mouse lungs in the immune system, innate immune response, inflammatory response, cytokine-mediated signal transduction, cellular response to interferon, and defense response to viruses. The DETs of the *T. gondii* RH infection group were significantly enriched in the B-cell receptor signaling pathway, sodium ion transport, and left/right axis specification, compared with the TGME49 infection group. Pathway analysis indicated that DETs were significantly enriched in protozoan, viral, and intracellular bacterial infections, cytokine-cytokine receptor interaction, and nucleotide-binding oligomerization domain (NOD)-like receptor (NLR) signaling pathway in mouse lungs infected with *T. gondii*.

### Changes in the expression of cytokines, chemokines, and inflammasomes

**Fig. 5:**
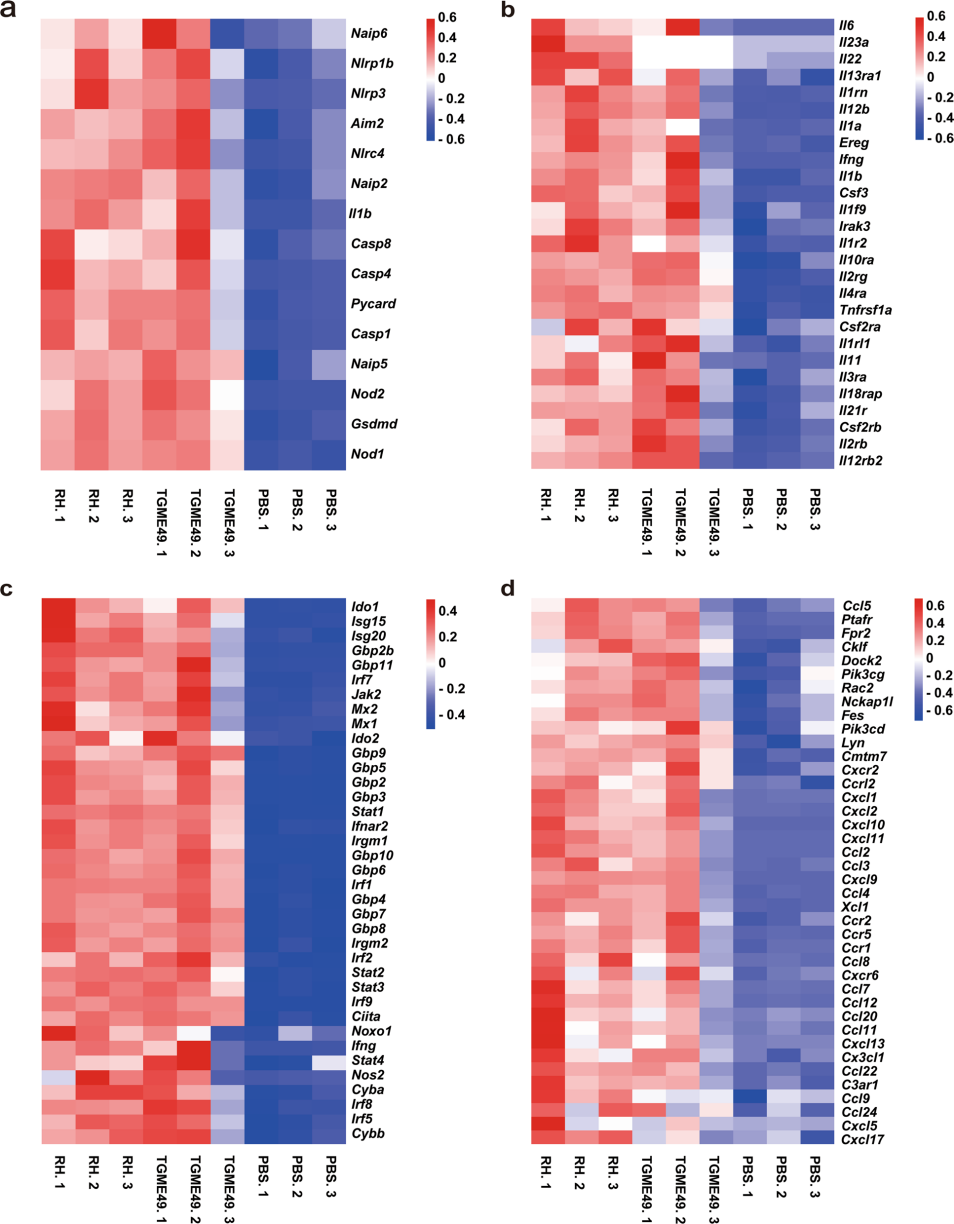
Heat map of inflammatory signaling molecules, cytokines, IFN-γ downstream effectors, and chemokines. Heat map of **a** inflammatory signaling molecules, **b** cytokines, **c** IFN-γ downstream effectors, and **d** chemokines

DETs were mainly enriched in inflammatory signaling pathways, chemokines, cytokines, and IFN-γ and its effector genes. Heat maps were generated for inflammatory signaling molecules and their downstream effectors. The transcription of inflammation-related factors absent-in-melanoma 2 (*Aim2*), *Nlrp1b*, *Nlrp3*, NRL family caspase activation and recruitment domain (CARD) domain-containing 4 (*Nlrc4*), NOD-like receptor family apoptosis inhibitory protein 2 (*Naip2*), *Naip6*, apoptosis-associated speck-like protein containing a CARD (*PYCARD*), caspase-1 (*Casp1*), *Casp4*, gasdermin-D (*Gsdmd*), and *Il1b* were significantly increased in the *T. gondii* infection group compared with the PBS control group (Fig. 5a). *Toxoplasma gondii* infection also significantly increased the transcription of *Ifng*, *Il1a*, *Il1b*, *Il6*, *Il10*, *Il11*, *Il12b,* and colony-stimulating factor 3 (*Csf3*) compared with that in the PBS control group. In addition, the transcription of *Il22* and *Il23* was significantly increased only in the *T. gondii* RH infection group (Fig. 5b). The increased transcription of *Ifng* induced the expression of *Ifng* downstream effector molecules (Fig. 5c). The expression of chemokines in the *T. gondii* infection groups, including *Ccl1*, *Ccl2*, *Ccl3*, *Ccl4*, *Ccl5*, *Ccl7*, *Ccl8*, *Ccl11*, *Ccl12*, *Ccl20*, *Ccl22*, *Ccl24*, C-X-C motif chemokine ligand 1 (*Cxcl1*), *Cxcl2*, *Cxcl5*, *Cxcl9*, *Cxcl10*, *Cxcl11*, and *Cxcl13* were significantly increased compared with those in the control group (Fig. 5d).

## Discussion

*Toxoplasma* pneumonia has become a serious opportunistic infection in AIDS and immunocompromised individuals [11]. Many studies have reported congenital toxoplasmosis, toxoplasmic encephalitis, and ocular toxoplasmosis; however, there are few studies on *Toxoplasma* pneumonia [12, 13]. In previous studies, mice were inoculated intraperitoneally with *T. gondii*, which can cause *Toxoplasma* pneumonia [14]. However, by establishing a mouse model of *Toxoplasma* pneumonia, multiple organs can be infected with *T. gondii* simultaneously. In this study, we developed a new method to establish a mouse model of *Toxoplasma* pneumonia caused by nasal droplet infection with *T. gondii*. Evans blue dye was simultaneously used to measure the function of vascular and epithelial barriers of murine lungs in vivo. It has also been used as a marker of albumin clearance in murine models of acute lung injury [15, 16]. In this study, we demonstrated that lung Evans blue staining could be used to assess the severity of air–blood barrier damage in *Toxoplasma* pneumonia. The results showed that lung Evans blue staining could be a crucial indicator of *Toxoplasma* pneumonia. Our approach thus provides a reliable infection model for the study of *Toxoplasma* pneumonia.

In this study, we compared the transcriptomes of lung tissues from C57BL/6 mice infected with *T. gondii* RH and TGME49 strains with those of PBS control mice using RNA-seq. DETs of 3160 and 1880 transcripts were altered during acute infection with *T. gondii* RH and TGME49, respectively. These enhanced transcriptome changes during *T. gondii* lung infection are consistent with previous results obtained from the transcriptome of *T. gondii* brain infections [17, 18]. Infection with *T. gondii* RH may result in greater changes in transcript abundance than infection with TGME49, reflecting a stronger host response to infection. Transcriptional analysis of mouse macrophages infected with different *T. gondii* strains revealed that host transcriptome expression was significantly different, although some common signals were similar [19]. Enrichment analysis of the transcripts was performed using the GO and KEGG databases to assess the biological relevance of the DETs. DETs were primarily associated with the immune system and microbial infections. The innate immune, inflammatory signaling, cytokine-mediated signaling, and chemokine signaling pathways were the major immune pathways with high gene enrichment. These results are similar to *T. gondii* infection of the brain [18, 20]. Transcriptome analysis of mouse lungs infected with *T. gondii* helped to understand the mechanism of the host immune system in *Toxoplasma* pneumonia.

Cytokines play a crucial role in host resistance to *T. gondii* infections. Many cytokines are induced by *T. gondii* infection [21]. IL-12, tumor necrosis factor alpha (TNF-α), and IFN-γ are important cytokines that are produced after *T. gondii* infection. Some results also reported that transcription of *Ifng*, *Il1a*, *Il1b*, *Il6*, *Il10*, and *Il12b* was significantly upregulated during *T. gondii* infection. Meanwhile, *Il11* was significantly upregulated in the lungs of mice infected with *T. gondii*. To the best of our knowledge, the upregulation of IL-11 in toxoplasmosis has never been reported before. IL-11 is involved in the induction of pulmonary fibrosis in lung diseases [22]. Based on our findings, we assumed that *T. gondii* infection of the lungs could induce pulmonary fibrosis; however, the exact mechanism requires further clarification. Notably, we found that the transcription of *Il22* and *Il23* was significantly upregulated only in the group infected with *T. gondii* RH. IL-23-dependent production of IL-22 is significantly upregulated and essential for the development of intestinal inflammation induced by *T. gondii* [23]. IL-22 and IL-23 may play an important role in *T. gondii* RH strain pneumonia and may be the reason for the strong pathogenicity of *T. gondii* RH strain compared with *T. gondii* TGME49 strain infections.

IFN-γ is a key cytokine for the control and elimination of *T. gondii* and is necessary for the host to resist infections [24]. At least four IFN-γ-mediated host mechanisms against *T. gondii* have been identified [10], of which IFN-γ-dependent cellular immunity plays an important role in the clearance of *T. gondii*. Additionally, IFN-γ can increase the expression of indoleamine 2,3 dioxygenase (IDO), which converts tryptophan, an essential amino acid for *T. gondii* development, into l-formyluridine. IFN-γ inhibits the growth and proliferation of *T. gondii* by inducing the mechanism of tryptophan degradation [25, 26]. Furthermore, IFN-γ can induce inducible nitric oxide synthase (iNOs), which increases the cellular concentration of NO. NO can inhibit *T. gondii* metabolic enzymes, resulting in *T. gondii* elimination [27]. Finally, IFN-γ-induced IRGs and GBPs lead to highly coordinated loading of *T. gondii* vacuoles, resulting in vacuole rupture and elimination of *T. gondii* via lysosomal-mediated degradation [28–30]. In this study, transcriptome analysis revealed that transcript levels of *Ifng*, *Ido1*, *Ido2*, *Nos2*, IRGs (*Irgm1*, *Irgm2*, *Irga6,* and *Irgb6*), and GBPs (*Gbp2*, *Gbp2b*, *GBP3*, *GBP4*, *Gbp5*, *Gbp6*, *Gbp7*, *Gbp8*, *Gbp9*, *Gbp10*, and *Gbp11*) were significantly upregulated during mouse lung infection with *T. gondii*, which is consistent with previous reports [18]. Notably, the transcription levels of *Isg15* were significantly upregulated during mouse lung infection with *T. gondii*. ISG15 links autophagy-mediated control to vacuole ubiquitination, which contributes to the elimination of *T. gondii* [31]. We also found that other IFN-induced effector genes (*Isg20*, *Mx1,* and *Mx2*) were significantly upregulated during lung infection. These effectors may play a pivotal role in the IFN-γ-induced elimination of *T. gondii*. Our data suggest that IFN-γ is a key cytokine for the control and elimination of *T. gondii* in *Toxoplasma* pneumonia.

Furthermore, studies have shown that inflammasomes play an important role in the pathogenesis of host infections caused by protozoan parasites [32]. A typical inflammasome consists of at least three major components: inflammatory cysteine aspartase (caspase-1, caspase-4, caspase-5, and caspase-11), an adaptor molecule PYCARD, and sensory proteins (NLRP1, NLRP3, NLRP6, NLRP12, NAIP1, NAIP2, NAIP5, NLRC4, AIM2, and pyrin) [33, 34]. In this study, we found that the transcription levels of *Nlrp1a*, *Nlrp1b*, *Nlrp3*, *Nlrc4*, *Aim2*, *Pycard*, *Casp1*, *Casp4*, pyroptosis executive molecule *Gsdmd*, and the proinflammatory cytokine *Il1b* were significantly upregulated in the lungs of mice infected with *T. gondii*. NLRP1 was involved in *T. gondii* pathogenesis. NLRP1-associated susceptibility alleles are directly associated with human congenital toxoplasmosis. Reduced *NLRP1* expression by monocytes results in the significantly reduced killing of *T. gondii* and increased cell death [35]. *Toxoplasma gondii* infection in murine bone marrow-derived macrophages can activate NLRP3 inflammasome and IL-1β. The expression of *Nlrp3* and *Il18* was increased in mice infected with *T. gondii*. Mice lacking *Nlrp1* also exhibited increased parasite burden and acute mortality [36]. Soluble total antigens derived from *T. gondii* tachyzoites stimulate THP-1 cells to increase the expression levels of *NLRP1*, *NLRP3*, *NLRC4*, and *AIM2* and release IL-1β [37]. Our study indicates that *T. gondii* lung infection can cause lung inflammation and induce pyroptosis. IL-1β helps the mice to build an immune response and contribute to *T. gondii* clearance.

In the immune system, chemokines guide immune effector cells to sites of infection or inflammation and coordinate interactions between immune cells [38]. In this study, the mRNA expression of *Ccl1*, *Ccl2*, *Ccl3*, *Ccl4*, *Ccl5*, *Ccl7*, *Ccl8*, *Ccl11*, *Ccl12*, *Ccl20*, *Ccl22*, *Ccl24*, *Cxcl1*, *Cxcl2*, *Cxcl5*, *Cxcl9*, *Cxcl10*, *Cxcl11,* and *Cxcl13* was significantly increased in the lungs of mice infected with *T. gondii*. Previous studies have shown that CCL1 and CCL22 promote Th2 chemotaxis migration [39, 40], whereas CCL7 and CCL12 promote the chemotactic migration of inflammatory monocytes [41, 42]. CCL3, CCL4, and CCL5 promote macrophage and natural killer (NK) cell chemotaxis [43, 44]. CCL11 and CCL24 promote inflammatory monocyte chemotaxis [45]. Other chemokines, such as CXCL1, CXCL2, and CXCL5, promote the chemotactic migration of neutrophils [46]. Furthermore, studies have shown that CXCL9, CXCL10, and CXCL11 promote the chemotactic migration of Th1 and B cells in combination with CXCL13 [38]. Mouse intestinal epithelial cells infected with *T. gondii* significantly increased mRNA expression of *Ccl2*, *Ccl3*, *Ccl5*, *Cxcl2*, *Cxcl9,* and *Cxcl10* [47]. In previous studies, the authors have used a BALB/c mouse model of toxoplasmic encephalitis. They demonstrated that the mRNA levels of *Cxcl9*, *Cxcl10*, *Cxcl11*, *Ccl2*, *Ccl3*, and *Ccl5* were significantly increased in the brain after *T. gondii* infection [48]. In another study, patients with ocular toxoplasmosis displayed high levels of IFN-induced chemokines CXCL9 and CXCL10 and circulating chemokines CCL25, CCL11, CXCL12, CXCL13, and CCL2 [49]. In this study, we found high mRNA levels of *Ccl24*, which has not been previously reported to be associated with *T. gondii* infection. CCL24 plays an important role in the pathological processes of skin and lung inflammation and fibrosis, and its antibody treatment can potentially be beneficial for therapeutic use in systemic sclerosis [50]. We propose that CCL24 may have a similar effect during *Toxoplasma* pneumonia; however, the precise mechanism still requires clarification. Our study also suggests that *Toxoplasma* pneumonia exhibits more complex chemokine expression than other *Toxoplasma* diseases. This result indicates that the degree of immune cell involvement in *Toxoplasma* pneumonia is complex.

## Conclusion

In this study, we established a new mouse model for *Toxoplasma* pneumonia and the first RNA-seq analysis of the transcriptome of lung tissues from C57BL/6 mice infected with *T. gondii* RH and TGME49 strains by nasal intubation drip. Our study revealed the functions of DETs in the lungs of mice infected with different *T. gondii* virulent strains. It was observed that lungs infected with *T. gondii* could activate host immune-related signaling pathways, including innate immune, inflammatory, cytokine-mediated, and chemokine signaling pathways to resist infection by *T. gondii*. We demonstrated that infection with the *T. gondii* RH strain produced greater changes in transcript abundance than *T. gondii* TGME49 strain infection, reflecting a stronger host response to infection. In addition, our results identified numerous new DETs during *Toxoplasma* pneumonia, expanding our knowledge of the host immune response and pathogenesis of *Toxoplasma* pneumonia.

## Supplementary Information

**Additional file 1: Table S1.** The primers used in the qRT–PCR analysis.

**Additional file 2: Table S2.** Differentially expressed transcripts (DETs) altered by *T. gondii* RH strain.

**Additional file 3: Table S3.** Differentially expressed transcripts (DETs) altered by *T. gondii* TGME49 strain.

**Additional file 4: Figure S1.** Verification of transcriptome accuracy by qRT–PCR. The **x**-axis represents the DETs, and the **y**-axis represents the relative expression of the gene. DETs above the horizontal line are upregulated and those below the horizontal line are downregulated. qRT–PCR: quantitative real-time PCR; DETs: differentially expressed transcripts.

**Additional file 5: Table S4.** Kyoto Encyclopedia of Genes and Genomes (KEGG) pathway enrichment analysis of the differentially expressed transcripts (DETs) altered by *T. gondii* RH strain.

**Additional file 6: Table S5.** Kyoto Encyclopedia of Genes and Genomes (KEGG) pathway enrichment analysis of the differentially expressed transcripts (DETs) altered by *T. gondii* TGME49 strain.

**Additional file 7: Table S6.** Gene Ontology (GO) enrichment analysis of the differentially expressed transcripts (DETs) altered by *T. gondii* RH strain.

**Additional file 8: Table S7.** Gene Ontology (GO) enrichment analysis of the differentially expressed transcripts (DETs) altered by *T. gondii* TGME49 strain.

## Data Availability

The datasets supporting the conclusions in this article are included within the article.

## Abbreviations

RNA-seq: RNA sequencing
dpi: Days post-infection
H&E: Hematoxylin and eosin
qRT–PCR: Quantitative real-time polymerase chain reaction
DETs: Differentially expressed transcripts
AIDS: Acquired immunodeficiency syndrome
IFN-γ: Interferon gamma
IL-12: Interleukin-12
TLRs: Toll-like receptors
GBPs: Guanylate-binding proteins
IRGs: Immunity-related GTPases
PV: Parasitophorous vacuole
CNS: Central nervous system
PBS: Phosphate-buffered saline
RPKM: Reads/kilobase/million mapped reads
FDR: False discovery rate
GO: Gene Ontology
KEGG: Kyoto Encyclopedia of Genes and Genomes
*Nod2*: Nucleotide-binding oligomerization domain-containing protein 2
*Irf5*: Interferon regulatory factor 5
*Ccl2*: C–C motif chemokine ligand 2
*Ciita*: Major histocompatibility complex (MHC) class II transactivator
*Nlrp1*: Nucleotide-binding domain-like receptor protein 1
BP: Biological process
CC: Cellular component
MF: Molecular function
*Cxcl1*: C-X-C motif chemokine ligand 1
*Csf3*: Colony-stimulating factor 3
IDO: Indoleamine 2,3-dioxygenase
iNOs: Inducible nitric oxide synthase
*Isg20*: Interferon-stimulated gene 20 kDa protein
*Mx1*: Myxovirus resistance 1
*Naip2*: NOD-like receptor family apoptosis inhibitory protein 2
*Nlrc4*: NRL family CARD domain-containing 4
*Aim2*: Absent-in-melanoma 2
