# Supplementary material for: Transcriptome analysis of a newly established mouse model of Toxoplasma gondii pneumonia
Source: Parasit Vectors. 2023 Feb 8;16:59. doi: 10.1186/s13071-022-05639-3 (PMC9906971; doi:10.1186/s13071-022-05639-3)
Supplement: Supplementary file 1 — Additional file 1: Table S1. The primers used in the qRT–PCR analysis. [file 13071_2022_5639_MOESM1_ESM.docx]

Table S1. The primers used in the qRT-PCR analysis.

| Gene Name | Forward Primer | Reverse Primer |
| --- | --- | --- |
| *Tlr7* | ATGTGGACACGGAAGAGACAA | GGTAAGGGTAAGATTGGTGGTG |
| *Tlr9* | ATGGTTCTCCGTCGAAGGACT | GAGGCTTCAGCTCACAGGG |
| *Nod2* | CAGGTCTCCGAGAGGGTACTG | GCTACGGATGAGCCAAATGAAG |
| *Ifng* | ATGAACGCTACACACTGCATC | CCATCCTTTTGCCAGTTCCTC |
| *Irf5* | AGAGACAGGGAAGTACACTGAAG | TGGAAGTCACGGCTTTTGTTAAG |
| *Ccl2* | TTAAAAACCTGGATCGGAACCAA | GCATTAGCTTCAGATTTACGGGT |
| *Ciita* | AGCAGGCCAAGACTTACATGA | CAGGCTGACATAGAGTCCTGT |
| *Nlrp1b* | TAGAAACGCCAGATAGGGTGA | AGTGTGATGGAAGTAATGGGGAT |
| *Cd36* | ATGGGCTGTGATCGGAACTG | GTCTTCCCAATAAGCATGTCTCC |
| *Il11* | TGTTCTCCTAACCCGATCCCT | CAGGAAGCTGCAAAGATCCCA |
| *Ccl24* | ATTCTGTGACCATCCCCTCAT | TGTATGTGCCTCTGAACCCAC |
| *Gapdh* | AGGTCGGTGTGAACGGATTTG | TGTAGACCATGTAGTTGAGGTCA |
