# Supplementary figures and images for: Transcriptome analysis of a newly established mouse model of Toxoplasma gondii pneumonia
Source: Parasit Vectors. 2023 Feb 8;16:59. doi: 10.1186/s13071-022-05639-3 (PMC9906971; doi:10.1186/s13071-022-05639-3)

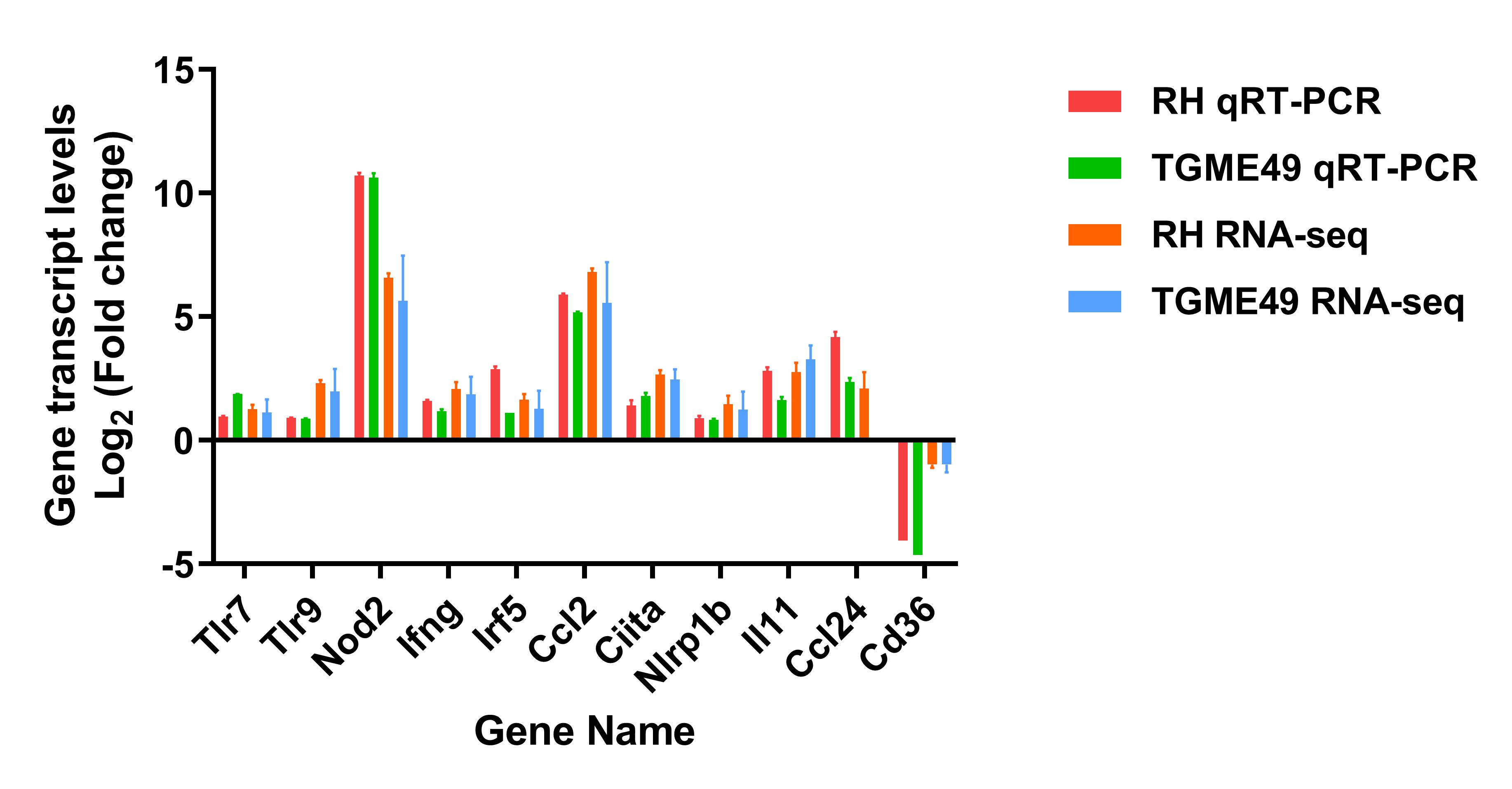

Supplement: Supplementary file 4 — Additional file 4: Figure S1. Verification of transcriptome accuracy by qRT–PCR. The x-axis represents the DETs, and the y-axis represents the relative expression of the gene. DETs above the horizontal line are upregulated and those below the horizontal line are downregulated. qRT–PCR: quantitative real-time PCR; DETs: differentially expressed transcripts. [file 13071_2022_5639_MOESM4_ESM.jpg]
